# Trends in industry payments and volume and distribution of robot-assisted surgeries

**DOI:** 10.1007/s00464-025-11724-2

**Published:** 2025-04-11

**Authors:** Katia Noyes, Ajay A. Myneni, Aaron B. Hoffman, Joseph D. Boccardo, Lorin M. Towle-Miller, Taylor Brophy, Steven D. Schwaitzberg

**Affiliations:** 1 Division of Health Services Policy and Practice, Department of Epidemiology and Environmental Health, School of Public Health and Health Professions, University at Buffalo, Buffalo, NY USA; 2 Department of Surgery, Jacobs School of Medicine and Biomedical Sciences, University at Buffalo, Buffalo, NY USA; 3 Department of Surgery, Methodist Dallas Medical Center, Dallas, TX USA; 4 Department of Biostatistics, School of Public Health and Health Professions, University at Buffalo, Buffalo, NY USA; 5 Department of Surgery, School of Medicine and Dentistry, University of Rochester, Rochester, NY USA

**Keywords:** Industry payments, Financial conflict of interest, Physician incentives, Robot-assisted surgery, Quality and safety

## Abstract

**Background:**

Considerable evidence links pharmaceutical industry payments to health providers’ over-prescribing behavior. In response, public policies were enacted to mitigate this effect. However, there is limited evidence examining surgical device industry payments and surgeons’ utilization of robot-assisted surgeries (RAS). This study assessed the relationship between industry payments to healthcare providers and the usage of RAS.

**Methods:**

Using 2015–2020 data from the CMS “Sunshine” Open Payments Database and New York State’s (NYS) Statewide hospital discharge databases, we assessed temporal and spatial relationships between industry payments to hospitals and surgeons and volumes of RAS.

**Results:**

During 2015–2020, general surgery robotic device manufacturers paid providers more than $236 M nationwide. The highest proportion of payments was made toward “Education and training” (66.6%) and “Food and travel” (20.6%). In NYS, gastrointestinal (GI) RAS volume steadily increased by 182% (2015–2019, *p* < 0.01), while there was a 150% increase in general surgeon payments. Genitourinary (GU) and gynecological (GYN) surgeon payments remained unchanged but GU and GYN RAS volume increased by 17% and 75%, respectively, during this period (*p* < 0.05). Approximately, 93% of payments and 98% of abdomen and pelvic RAS in NYS were concentrated in metropolitan or non-rural counties.

**Conclusions:**

With increasing payments from robotic device companies toward surgeon education and training, the use of RAS is likely to continue to rise in the long term. Unbiased and non-industry-funded studies examining RAS effect on surgeon behavior and patient outcomes are imperative to ensure system efficiency and patient safety.

**Supplementary Information:**

The online version contains supplementary material available at 10.1007/s00464-025-11724-2.

Strong empirical evidence has demonstrated that industry payments influence physician behavior and treatment decision-making [1, 2]. Such payments may come in many forms, including kickbacks, consultant fees, royalties, clinical trial funding, honorariums, meals and travel expenses, speaker’s fees, and educational and research grants. Prescribing practices may not only be influenced by direct payments to physicians, such as dinners and gifts, but also by sponsored programs and activities that physicians are invited to attend, including lectures and industry-sponsored Continuing Medical Education (CME) activities [3].

To mitigate this payment-related conflict of interest bias, numerous policies have been adopted by government and professional organizations to inform the public about financial relationships between industry and healthcare providers, and to penalize pharmaceutical corporations for providing misleading information and financial incentives to physicians to engage in inappropriate prescribing [4]. As part of the Affordable Care Act, the Physician Payments Sunshine Act of 2010 requires medical device and pharmaceutical companies to report all payments given to providers and facilities, beginning in 2013. The Act mandates that companies report all payments on an annual basis. All industry payments to physicians and teaching hospitals are made publicly available in the Open Payments database (OPD) in an effort to “help prevent inappropriate influence on research, education and clinical decision making” [5, 6].

While the pharmaceutical industry’s influence over prescriptions has been the primary focus of these public disclosure policies for over a decade now, surgical device manufacturers continue using similar tactics. One example is robot-assisted surgery (RAS), where the entire surgical field has been introduced to new technology mainly through marketing practices within a short time period, in the absence of any evidence supporting RAS superiority over open or laparoscopic approaches. Use of RAS has continued to rise in the presence of recent controversial empirical evidence on post-RAS patient outcomes, with well-documented higher costs of RAS [7–9].

This study examined reported industry payments from general surgery robotics (GSR) companies and their allocation toward different payment categories. Further the relationship between industry payments to individual surgeons and hospitals and the use of robotic surgery in three clinical areas where RAS has been the most prevalent: urology, gynecology, and general (including gastrointestinal) surgery were examined. Lastly, the trends over time and variation by surgeon specialty and geographic regions to elucidate mechanisms behind these relationships were also examined.

## Methods

### Data sources

#### Open payments database (OPD)

Use of 2015–2019 data from the NYS Statewide Planning and Research Cooperative System (SPARCS), an all-payer data reporting system that includes patient characteristics, diagnoses, and treatments, was guided by the Data Use Agreement with NYS Department of Health and approved by University at Buffalo’s Institutional Review Board (UB IRB). Data from 2020 were not included considering the unexpected disruption in surgical volume during the peak of SARS CoV2 pandemic. We did not seek UB IRB’s approval for use of data from the OPD, as it is a publicly available database.

#### Payments to physicians and teaching hospitals

The OPD was used to quantify industry-funded payments from GSR companies made to hospitals and physicians from 2015 to 2020. Categories of reported payments were re-categorized into five groups, including “Education and training” (includes “Education,” “Services other than consulting,” and “Grant” payments), “Travel and food” (includes “Travel and lodging” and “Food and beverage” payments), “Direct payments” (includes “Gift,” “Charitable contribution,” “Honoraria,” “Consulting fee,” and “Royalty and License” payments), “Space rental and facility fee” and “Research.” The last two categories apply to teaching hospitals only. “Current or prospective ownership or investment interests” payments from OPD were not included as this category was only added in 2019.

To examine the relationship between industry payments and surgical volume, the dataset was limited to only payments made to NYS surgeons performing abdomen/pelvic robotic surgeries, including gastrointestinal (GI), genitourinary (GU), and gynecologic (GYN) procedures from the OPD. We included physician specialties of “Surgery,” Colon and Rectal Surgery,” “Surgical Oncology,” “Gynecology,” “Gynecological Oncology,” and “Urology” to identify payments made to surgeons performing abdomen and pelvic RAS procedures.

#### Utilization of robot-assisted surgeries

Surgeon and hospital volumes of abdominal/pelvic RAS procedures performed between 2015 and 2020 were calculated by identifying these procedures using ICD and CPT codes. To identify robot-assisted procedures, ICD-9 code 17.41, 17.42, 17.43, 17.44, and 17.49; ICD-10 codes (8E0W0CZ, 8E0W4CZ) and CPT code (S2900) were used.

#### Revenue of GSR companies

Annual revenues (in USD) of the GSR companies for the years 2015 to 2020 were procured from the annual reports to the Securities and Exchange Commission [10–15].

### Statistical analysis

Using data from SPARCS and OPD, trends in volumes of RAS and surgeon payments for each year in NYS between 2015 and 2020 were analyzed. The volumes of the abdominal/pelvic RAS procedures by the rurality status (rural/urban) of the facility where the surgery was performed were also examined. Rurality was defined as a dichotomous variable (rural, yes or no) based on data provided by the U.S. Census Bureau and designation by New York State Housing Coalition, where more than 50% of the population living in rural areas as rural and those with more than 50% of the population living in urban or metropolitan regions as urban counties [16].

**Fig. 1:**
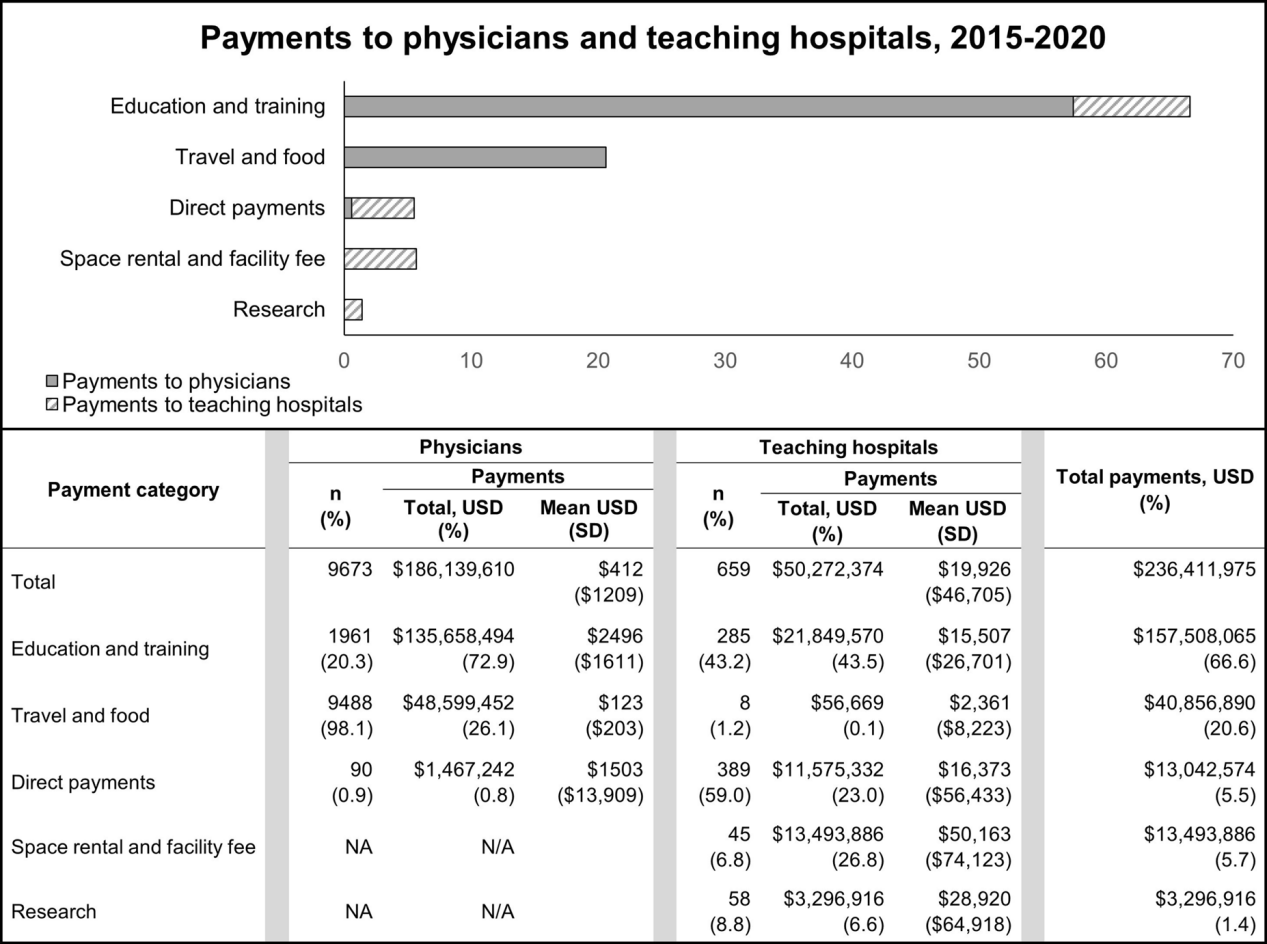
Payments reported in the Open Payments database between 2015 and 2020. The plot shows the breakdown of the payments to physicians and teaching hospitals among the major categories as a percentage of total payments in each category. The solid section of the plot represents the payments to physicians, and the patterned sections represent those made to teaching hospitals. The whole bar represents the total payments in each category. The table below the plot shows details of the number of physicians and teaching hospitals who received payments, the total, mean, and standard deviation of the payments in each category in US dollar (USD). The percentages in parentheses reflect the proportion of the overall total payments to both physicians and teaching hospitals—$236,411,975

**Fig. 2:**
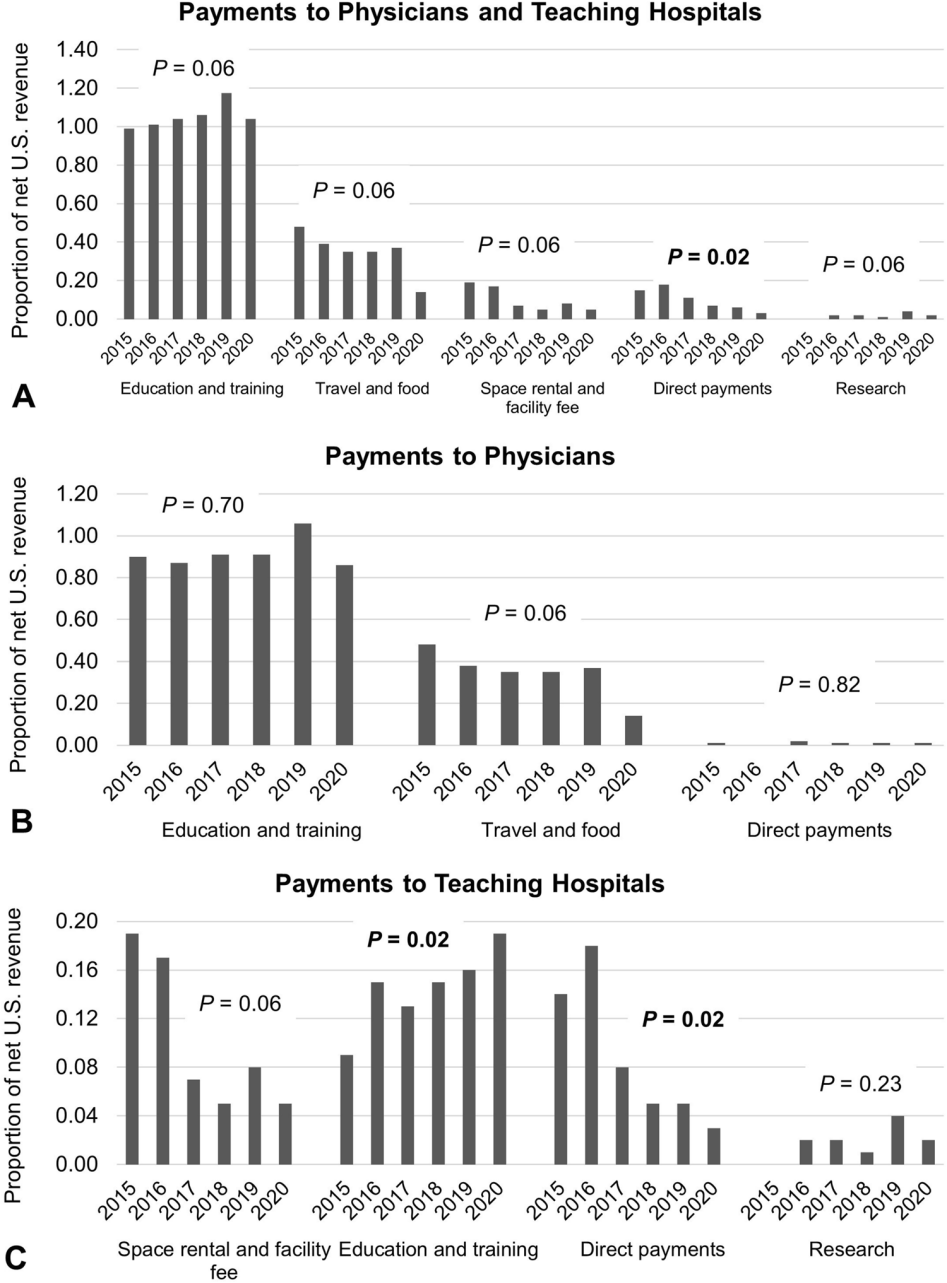
Plots showing annual payments to physicians and teaching hospitals combined (**A**), physicians (**B**), and teaching hospitals (**C**) among individual categories as a proportion of net U.S. revenue reported by general surgery robotics companies between 2015 and 2020. We did not include the categories where payments were less than 0.0% of the net revenue

The payments to physicians and teaching hospitals were examined by plotting them as a proportion of total payments among the five categories chosen for analysis (Fig. 1) and among all categories reported in OPD (Supplementary Table 1). Additionally, the linear trend of payments (2015–2020) was also examined by plotting them as a percentage of annual net revenue of GSR companies in the US [17–22] (Fig. 2A, B, and C).

**Fig. 3:**
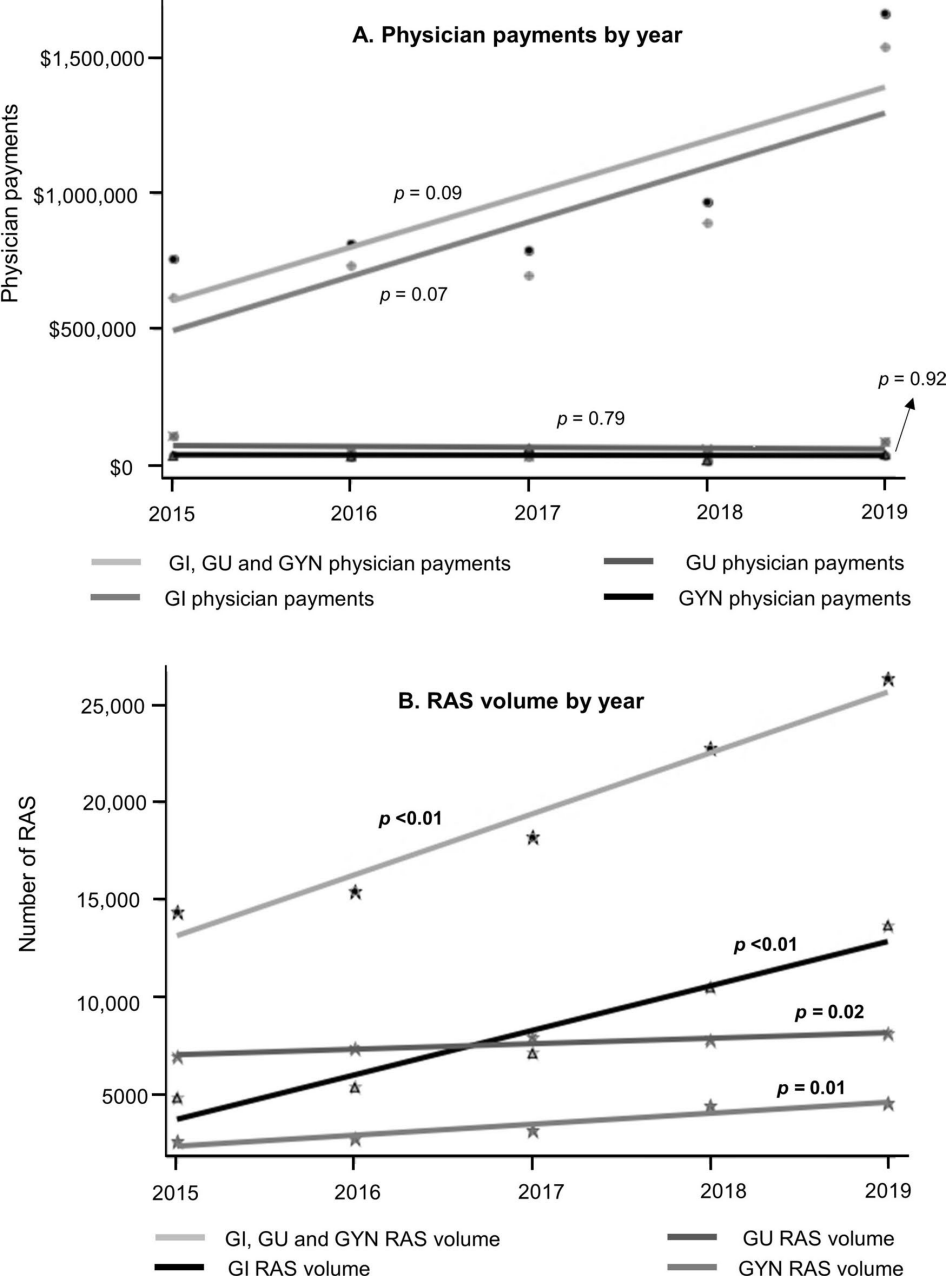
Plot showing payments to physicians (**A**) performing robot-assisted procedures (RAS) and corresponding volume of these procedures (**B**) in New York between 2015 and 2020. *GI* gastrointestinal, *GU* genitourinary, *GYN* gynecological

In NYS, annual trends were tracked in payments to all surgeons performing abdomen/pelvic RAS as well as those performing gastrointestinal (GI), gynecological (GYN), and genitourinary (GU) RAS, along with the corresponding volume of RAS in those categories performed during the study period (Fig. 3).

**Fig. 4:**
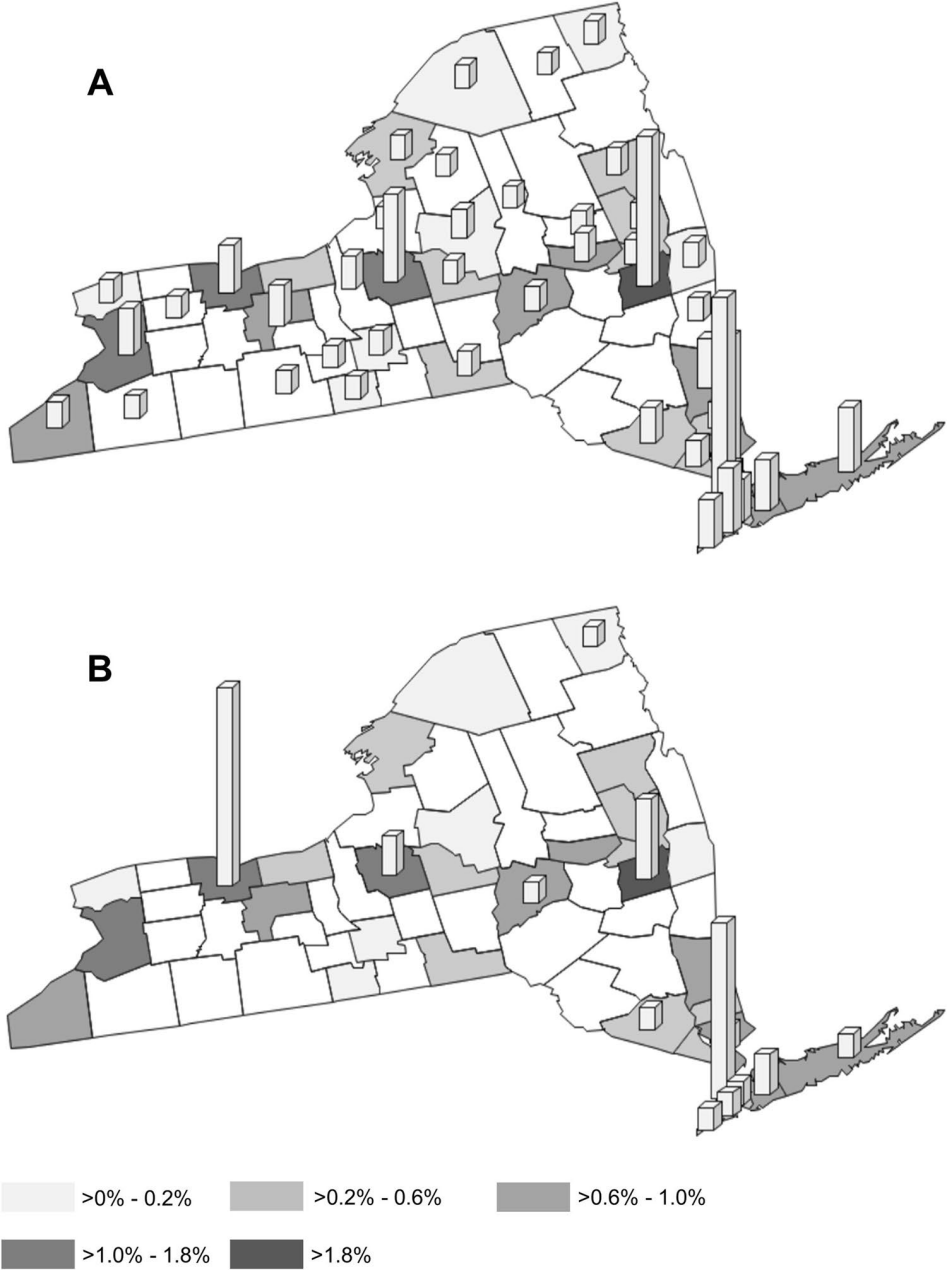
Map plots showing the proportion of abdomen and pelvic robot-assisted surgery procedures in New York State among major metropolitan counties versus rural counties between 2015 and 2020. Vertical bars represent total payments during the same period physicians (**A**) and teaching hospitals (**B**)

Lastly, the payments to physicians and teaching hospitals and the combined payments for abdomen/pelvic RAS as well as volume of RAS in NYS were examined by plotting them on a county level (Fig. 4). The relationships of average RAS payments and volumes with county rurality (urban/rural) were assessed using two sample *t* tests.

Alpha for statistical tests was set at 0.05. All analyses were conducted using Microsoft Excel 2019 and SAS 9.4 (SAS Institute, Cary, NC).

## Results

### Overview of the industry spendings

Figure 1 describes the industry payments to surgeons and teaching hospitals by payment category. About three quarters of the overall payment ($236,411,975) was paid to 9673 physicians ($186,139,610, 79%), while 659 teaching hospitals received $50,272,374 (21%). For physicians, most payments were in “Education and training” ($135,658,494, 73%) and “Travel and food” ($48,599,452, 26%) categories, with “Education and training” payments being on average higher (mean: $2496, standard deviation, SD: $1611) than “Direct physician payments” (mean: $1503, standard deviation, SD: $13,909). For teaching hospitals, most payments were in “Education and training” ($21,849,571, 43%) and “Space rental and facility fee” ($13,493,886, 27%) categories, with higher average payments for “Space rental and facility fee” (mean: $50,163, SD: $74,123) and “Research” (mean: $28,920, SD: $64,918) categories. A more detailed breakdown of the payments to physicians and teaching hospitals among all categories is found in Supplementary Table 2.

### Payments trend over time

Figure 2 illustrates the payments to physicians and teaching hospitals as a proportion of GSR companies’ reported annual US revenue. Overall, the proportion of total revenue (< 1.3%) toward payments to physicians and teaching hospitals remained steady except for “Direct payments,” which showed a decreasing trend (*p* = 0.02) from 2015 to 2020. Payments to physicians ranged from < 0.01% (in “Direct payments”) to 1.06% (in “Education and training”) of net US revenue and did not change during the study period. Payments to teaching hospitals ranged from 0.01% (in “Research”) to 0.19% (in “Education and training” and “Space rental and facility fee”) of net US revenue and payments in “Education and Training” showed an increasing trend (*p* = 0.02), while payments in “Direct payments” showed a decreasing trend over time (*p* = 0.02).

### Relationship between industry payments and surgical volumes

Payments made to physicians performing RAS and number of abdomen/pelvic RAS (GI, GU, GYN, and total trunk) performed between 2015 and 2019 are presented in Fig. 3. While general surgeon payments did not significantly increase between 2015 and 2019 (*p* = 0.07), the volume of GI RAS increased steadily (*p* < 0.01). Payments to GU and GYN surgeons remained stable. However, the surgical volume in both GU (*p* = 0.02) and GYN (*p* = 0.01) showed an upward trend.

Most of the abdomen and pelvic RAS procedures were performed in urban counties (5472 procedures a year on average among urban county vs. 46 procedures a year among rural counties; *p* < 0.01, Fig. 4). Payments made to surgeons and teaching hospitals were also concentrated in the urban counties ($146,177 per year on average in urban counties vs. $3,636 per rural county; *p* < 0.01).

## Discussion

In this population-based study, we examined the association between surgical device industry payments to providers and RAS volumes. Our results demonstrated a significant positive correlation between industry payments and volumes of RAS performed. Three quarters of the overall industry funding went to support education activities and surgeons’ travel to training and conferences, with less than 1% of the overall funding going to support research initiatives. Our spatial analysis demonstrated that the industry payments are concentrated in large academic health centers. In examining data provided by GSR companies to the OPD database from 2015 through 2020, we demonstrated that most of the industry payments went to individual physicians (78.7%) as opposed to teaching hospitals (21.3%). As GSR companies’ revenue in the US increases, the total amount of outgoing payments has remained stable.

The development of robotic surgical approach in the early 2000s has created a fundamental shift in surgical practice, similar to the introduction of laparoscopic surgery in the 1980s. Most current evidence on RAS effectiveness and outcomes in general surgery for GI procedures comes from non-inferiority studies of RAS compared to laparoscopic surgery. Despite similar safety profile, RAS procedures were shown to be associated with greater risks of prolonged operative time, the financial burden of increased instrument cost, and a need for adequate learning curve of both surgical and peri-operative providers [23, 24]. A significant portion of the appeal of RAS in general surgery has been due to surgeons perception of greater ergonomics of RAS and patient preference for innovative medical technology [25, 26]. As a result, the field of general surgery is seeing rapid adoption of RAS despite higher operating costs of robotic platform [7, 9, 27, 28].

Our research demonstrated a positive correlation between industry payments and volume of RAS performed and time trends further suggest a causal relationship. We found that both use of robotics in GI procedures and payments to physicians performing GI surgeries increased dramatically ranging from $617,811 in 2015 to $1,542,619 in 2019. Similarly, studies reported a 30% rise in RAS volume in the US in 2019 alone [29]. We hypothesize that investing in payments to general surgeons and teaching hospitals is used by the device industry as a dissemination and scale-up strategy aimed at expanding RAS usage in procedures where clinical benefits are equivocal.

For GYN and GU procedures, the current findings indicate that payments remained unchanged between 2015 and 2019, but volume of RAS increased in the same period; payments to physicians doing GYN ($142,198 in 2015 to $38,657 in 2019) and GU ($107,602 in 2015 to $85,527 in 2019) RAS decreased and were much lower. Historically, since the initial implementation of RAS in GU and GYN surgical fields several decades prior, there has been a marked improvement in patient outcomes after RAS when compared to the open proctectomy and hysterectomy [22, 30, 31]. Because of the more established advantages that RAS offers in GU and GYN compared to open procedures, it is plausible that there is less of a need for device companies to incentivize physicians in these specialties, as use of RAS for these procedures will likely continue to rise regardless of industry funding.

A notable finding of the current study is the small proportion of industry payments allocated to research, comprising only 1.4% of total payments or 0.02% of the companies’ annual US revenue. As a reference, previous analyses have demonstrated that large medical device companies spend between 5 and 15% of annual revenue on research and development of their products [22]. More technologically dependent hardware, such as implantable pacemakers, tending to be at the higher end of this range, while more simple devices, such as prosthetic joints, tend to be at the lower end [19]. Considering the extremely rapid growth of robotic surgical industry and the subsequent widespread rollout of this technology in clinical care, it is not clear why such a small percentage of revenue is allocated to research including studies of comparative effectiveness of RAS vs other alternatives, surgeon preferences, long-term post-surgical patient safety and outcomes.

It is possible that demand for industry-sponsored research has declined due to better transparency and understanding of bias in research. It has been demonstrated that researchers funded by industry reliably publish findings favorable to their sponsors [19]. On the contrary, pharmaceutical and medical device research tends to be extremely expensive, and would be virtually impossible without companies investing in a product’s development [21]. While there is considerable concern for bias in industry-funded research, non-biased research should be in the best interest of a company to identify the most appropriate role for a given product or therapeutic.

While adequately training physicians is essential to promote safe and effective use of emerging medical technology, especially for surgical devices, our results suggest that this may also be influencing practice patterns of surgeons toward a greater use of emerging technology, regardless of the state of research evidence. This is similar to the trends observed earlier with pharmaceutical companies’ sponsoring of CME events, which resulted in greater use of brand name drugs, as well as increased off-label use of these medications [20].

Finally, this study revealed that payments from GSR companies and performance of RAS procedures are concentrated heavily in the metropolitan area of NYS. We found that 98% of all RAS cases and 93% of physician payments occurred in urban hospitals and surgeons who practice there, while at least 10% of NYS population resides in rural counties. This clearly indicates that patients receiving care in rural centers are less likely to receive RAS across all indications. One possible explanation is that RAS is a more costly option than laparoscopic surgery from the hospital perspective and requires a large volume of robotic cases to improve return on investment, which is often not feasible in rural hospitals [17]. At the same time, in rural communities where physician shortages remain a major barrier to healthcare access, RAS may be especially useful tool because it may obviate the need for an assistant surgeon [32, 33]. Time required to train rural surgeons who lack RAS training is another limiting factor. Rural surgeons may be limited to a range of only hours per month, making adoption and training for RAS less feasible in these regions [18, 34].

The OPD is a publicly available database comprised of mandatory reported monetary transactions from industry to physicians and teaching hospitals in the United States. Since its implementation following the passage of the Physician Payments Sunshine Act in 2010, OPD has prompted closer public attention of monetary transactions between industry and physicians [35–37]. This legislation has led to recent large-scale lawsuits over failure to disclose financial conflicts of interest [38]. However, several concerns about the implementation of the Sunshine Act limit the potential impact of this legislation. Whether intentional or not, there is still widespread failure of authors to accurate disclose conflicts of interest, particularly with GSR companies [39]. Physicians who receive smaller payments, such as food and travel expenses, tend to underreport monetary interactions in their publication disclosures when compared to payments reported in the OPD. Conversely, ownership of stocks and the profits associated with such have not been consistently reported to the OPD, but they are often reported by physicians when presenting research. This group of physicians tends to over-report financial disclosure when compared to payments reported in the OPD. The OPD only recently introduced a new payment category of ‘Current or prospective ownership and investment interests’ in 2022; however, these payment categories only apply to payments made starting in 2018 to present and were not included in the current analysis. Providers can review and request corrections to all reported data. However, many providers do not review the information provided by industry, which can lead to discordance between industry and physician reporting.

The limitations of the CMS OPD dataset explain several constraints of this study. First, because industry payments were reported annually as a total amount, we could not examine the causal relationship between these payments and surgeon behavior. Additionally, industry-sponsored training often covers a broad range of topics related to surgical skills, safety, and technology use, not just for surgeons, but also for other healthcare providers and hospital staff. Finally, due to hospital mergers and acquisitions, many surgical training programs now serve multiple hospitals and provide training to a wider pool of providers, including surgeons in rural areas.

## Conclusion

With substantial industry payments continuously being allocated to surgical education and training of physicians, the use of RAS will certainly continue to rise in the long term. Despite significant investment in surgeon RAS education and training, a miniscule percentage of industry payments (1.4%) had been allocated to furthering comparative research on RAS outcomes undermining quality improvement efforts and ability to track patient safety and provider preferences.

## Supplementary Information

Below is the link to the electronic supplementary material.

Supplementary file1 (DOCX 31 KB)

## References

[1] Robertson C, Rose S, Kesselheim AS (2012) Effect of financial relationships on the behaviors of health care professionals: a review of the evidence. J Law Med Ethics 40:452–466 23061573 10.1111/j.1748-720X.2012.00678.x

[2] Wazana A (2000) Physicians and the pharmaceutical industry: is a gift ever just a gift? JAMA 283:373–380 10647801 10.1001/jama.283.3.373

[3] Steinman MA, Boscardin CK, Aguayo L, Baron RB (2010) Commercial influence and learner-perceived bias in continuing medical education. Acad Med 85:74–79 20042828 10.1097/ACM.0b013e3181c51d3f PMC2801075

[4] Office of Public Affairs (2020) Justice department announces global resolution of criminal and civil investigations with opioid manufacturer purdue pharma and civil settlement with members of the sackler family. Office of Public Affairs, U.S Department of Justice, Washington

[5] U.S. Centers for Medicare and Medicaid Services (2023) Open payments. U.S. Centers for Medicare and Medicaid Services, Baltimore, MD 26110197

[6] Kirschner NM, Sulmasy LS, Kesselheim AS (2014) Health policy basics: the physician payment sunshine act and the open payments program. Ann Intern Med 161:519–521 25069795 10.7326/M14-1303

[7] Han C, Shan X, Yao L, Yan P, Li M, Hu L, Tian H, Jing W, Du B, Wang L, Yang K, Guo T (2018) Robotic-assisted versus laparoscopic cholecystectomy for benign gallbladder diseases: a systematic review and meta-analysis. Surg Endosc 32:4377–4392 29956028 10.1007/s00464-018-6295-9

[8] Jayne D, Pigazzi A, Marshall H, Croft J, Corrigan N, Copeland J, Quirke P, West N, Edlin R, Hulme C (2019) Robotic-assisted surgery compared with laparoscopic resection surgery for rectal cancer: the ROLARR RCT. Effic Mech Eval. 10.3310/eme06100 31556981

[9] Sheetz KH, Dimick JB (2019) Is it time for safeguards in the adoption of robotic surgery? JAMA 321:1971–1972 31038659 10.1001/jama.2019.3736 PMC7032039

[10] United States Securities and Exchange Commission (2016) Intuitive Surgical, Inc., SEC

[11] United States Securities and Exchange Commission (2017) Intuitive Surgical, Inc., SEC

[12] United States Securities and Exchange Commission (2018) Intuitive Surgical, Inc., SEC

[13] United States Securities and Exchange Commission (2019) Intuitive Surgical, Inc., SEC

[14] United States Securities and Exchange Commission (2020) Intuitive Surgical, Inc., SEC

[15] United States Securities and Exchange Commission (2021) Intuitive Surgical, Inc., SEC

[16] Borges MS (2023) A report on recent changes in the population, demographics, housing, and income characteristics of Rural New York State (2011–2020) State of Rural New York, New York State Rural Housing Coalition, Albany

[17] Brinkman W, de Angst I, Schreuder H, Schout B, Draaisma W, Verweij L, Hendrikx A, van der Poel H (2017) Current training on the basics of robotic surgery in the Netherlands: time for a multidisciplinary approach? Surg Endosc 31:281–287 27194262 10.1007/s00464-016-4970-2 PMC5216079

[18] Carpenter BT, Sundaram CP (2017) Training the next generation of surgeons in robotic surgery. Robot Surg 4:39–44 30697562 10.2147/RSRR.S70552 PMC6193443

[19] Criss CN, Gadepalli SK (2018) Sponsoring surgeons: an investigation on the influence of the da Vinci robot. Am J Surg 216:84–87 28859920 10.1016/j.amjsurg.2017.08.017

[20] Fugh-Berman A (2021) Industry-funded medical education is always promotion-an essay by Adriane Fugh-Berman. BMJ 373:n1273 34088736 10.1136/bmj.n1273

[21] Goold SD, Campbell EG (2008) Industry support of continuing medical education: evidence and arguments. Hastings Cent Rep 38:34–37 19192715 10.1353/hcr.0.0084

[22] The Medicare Payment Advisory Commission (2017) An overview of the medical device industry. The Medicare Payment Advisory Commission, Washington, pp 207–242

[23] Paraiso MF, Ridgeway B, Park AJ, Jelovsek JE, Barber MD, Falcone T, Einarsson JI (2013) A randomized trial comparing conventional and robotically assisted total laparoscopic hysterectomy. Am J Obstet Gynecol 208(368):e361-367 10.1016/j.ajog.2013.02.008 23395927

[24] Rosemurgy A, Ryan C, Klein R, Sukharamwala P, Wood T, Ross S (2015) Does the cost of robotic cholecystectomy translate to a financial burden? Surg Endosc 29:2115–2120 25492447 10.1007/s00464-014-3933-8

[25] Geller EJ, Matthews CA (2013) Impact of robotic operative efficiency on profitability. Am J Obstet Gynecol 209(20):e21-25 10.1016/j.ajog.2013.03.030 23535238

[26] Irani M, Prabakar C, Nematian S, Julka N, Bhatt D, Bral P (2016) Patient perceptions of open, laparoscopic, and robotic gynecological surgeries. Biomed Res Int 2016:4284093 27840826 10.1155/2016/4284093 PMC5093233

[27] Sheetz KH, Claflin J, Dimick JB (2020) Trends in the adoption of robotic surgery for common surgical procedures. JAMA Netw Open 3:e1918911 31922557 10.1001/jamanetworkopen.2019.18911 PMC6991252

[28] Rizzo KR, Grasso S, Ford B, Myers A, Ofstun E, Walker A (2023) Status of robotic assisted surgery (RAS) and the effects of coronavirus (COVID-19) on RAS in the department of defense (DoD). J Robot Surg 17:413–417 35739435 10.1007/s11701-022-01432-7 PMC9225798

[29] Buderath P, Aktas B, Heubner M, Kimmig R (2015) Robot-assisted hysterectomy: a critical evaluation. Robot Surg Res Rev 2:51–58

[30] Martino MA, Berger EA, McFetridge JT, Shubella J, Gosciniak G, Wejkszner T, Kainz GF, Patriarco J, Thomas MB, Boulay R (2014) A comparison of quality outcome measures in patients having a hysterectomy for benign disease: robotic vs. non-robotic approaches. J Minim Invasive Gynecol 21:389–393 24513969 10.1016/j.jmig.2013.10.008

[31] McAlpine K, Forster AJ, Breau RH, McIsaac D, Tufts J, Mallick R, Cagiannos I, Morash C, Lavallee LT (2019) Robotic surgery improves transfusion rate and perioperative outcomes using a broad implementation process and multiple surgeon learning curves. Can Urol Assoc J 13:184–189 30407153 10.5489/cuaj.5527 PMC6570603

[32] Alligood-Percoco NR, Huggler AD, McQuillen AN (2023) Implementation of a robotic gynecologic surgery program in a rural setting: impact on presence of assistant surgeon and route of hysterectomy. JSLS. 10.4293/JSLS.2023.00035 37746520 10.4293/JSLS.2023.00035 PMC10516264

[33] Larson E, Andrilla C, Kearny J, Garberson L, Patterson D (2021) The distribution of the general surgery workforce in rural and urban America in 2019. Policy. Brief WWAMI rural health research center. University of Washington, Washington

[34] Mikhailova O (2018) Adoption and implementation of new technologies in hospitals: a network perspective. IMP J. 10.1108/IMP-05-2017-0027

[35] DeFrance MJ, Yayac MF, Courtney PM, Squire MW (2021) The impact of author financial conflicts on robotic-assisted joint arthroplasty research. J Arthroplast 36:1462–1469 10.1016/j.arth.2020.10.033 33199093

[36] Hase NE, Passarelli J, Robichaud S, Segalini N, Ganguli S, Song C, Rabinowitz J, Aziz H (2022) The undisclosed disclosures: conflicts of interest in studies related to robotics in hepatobiliary and pancreatic surgery. Surgery 172:1429–1433 36096965 10.1016/j.surg.2022.08.013

[37] Karamchandani MM, Tian T, Hall R, Nickel I, Aalberg J, Lassaletta AD, Chatterjee A, Walters DM (2024) Discrepancies in financial conflicts of interest in robotic cardiothoracic surgery studies. Ann Thorac Surg 117:466–472 37271443 10.1016/j.athoracsur.2023.04.047

[38] Office of Public Affairs (2020) Medtronic to pay over $9.2 million to settle allegations of improper payments to South Dakota neurosurgeon. U.S. Department of Justice, Washington

[39] Myneni AA, Brophy T, Harmon B, Boccardo JD, Burstein MD, Schwaitzberg SD, Noyes K, Hoffman AB (2023) The impact of disclosure of conflicts of interest in studies comparing robot-assisted and laparoscopic cholecystectomies-a persistent problem. Surg Endosc 37:1515–1527 35851821 10.1007/s00464-022-09440-2

