# Supplementary material for: Trends in industry payments and volume and distribution of robot-assisted surgeries
Source: Surg Endosc. 2025 Apr 11;39(5):3215–23. doi: 10.1007/s00464-025-11724-2 (PMC12041063; doi:10.1007/s00464-025-11724-2)
Supplement: Supplementary file 1 — Supplementary file1 (DOCX 31 KB) [file 464_2025_11724_MOESM1_ESM.docx]

**Supplementary Table 1**. Payment categories as described in the Open Payments database and regrouped categories as used in the current study.

| **Regrouped payment categories** | **Payments categories in the Open Payments database** | **Definitions from Open Payments database** |
| --- | --- | --- |
| Education and training | Education | Payments or transfers of value for classes, activities, programs or events that involve the imparting or acquiring of particular knowledge or skills, such as those used for a profession. This category can include things like textbooks and medical journal articles |
|  | Services other than consulting, including serving as faculty or as a speaker at a venue other than a continuing education program | Payments made to physicians for speaking, training, and education engagements that are not for continuing education. |
|  | Grant | Payments to a physician or teaching hospital in support of a specific cause or activity |
|  |  |  |
|  |  |  |
| Travel and Food | Travel and lodging |  |
|  | Food and beverage |  |
|  |  |  |
|  |  |  |
| Space rental and facility fee | Space rental and facility fee | Fees for renting space or facilities, in a teaching hospital, for example |
|  |  |  |
|  |  |  |
| Direct payments | Gift | A general category, which often includes anything provided to a physician or teaching hospital that does not fit into another category |
|  | Charitable contribution | Any payment or transfer of value made to an organization with tax-exempt status under the Internal Revenue Code of 1986, but only if it is not more specifically designed by one of the other nature or payment categories |
|  | Honoraria | Similar to consulting fees, but generally reserved for a one-time, short duration activity. Also distinguishable in that they are generally provided for services which custom prohibits a price from being set. |
|  | Consulting fee | Payments made to physicians for advice and expertise on a particular medical product or treatment, typically provided under a written agreement and in response to a particular business need. These payments often vary depending on the experience of the physician being consulted. |
|  | Royalty or License | Royalty or other payment based on sales of products that use a physician’s intellectual property |
|  |  |  |
|  |  |  |
| Research | Research | Funding for a research project of study where the physician is named as a principal investigator |

**Supplementary Table 2**. Payments reported between 2015-2020 to physicians, teaching hospitals, and total amounts.

| **Payment category** | **Physicians**  **USD (%)** | **Teaching Hospitals**  **USD (%)** | **Total**  **USD (%)** |
| --- | --- | --- | --- |
| **Total** | **$186,139,601 (78.7)** | **$50,272,374 (21.3)** | **$236,411,975 (100)** |
|  |  |  |  |
| Education and training | $135,658,494 (57.4) | $21,849,570 (9.2) | $157,508,065 (66.6) |
| Education | $116,458,299 (49.3) | $18,113,951 (7.7) | $134,572,250 (56.9) |
| Services other than consulting | $19,200,195 (8.1) | $2,664,242 (1.1) | $21,864,437 (9.2) |
| Grant | N/A | $1,071,377 (0.5) | $1,071,377 (0.5) |
|  |  |  |  |
| Travel and food | $48,599,452 (20.6) | $56,669 (0.0) | $48,656,121 (20.6) |
| Travel and lodging | $34,683,377 (14.7) | $85 (0.0) | $34,683,462 (14.7) |
| Food and beverage | $13,916,075 (5.9) | $56,584 (0.0) | $13,972,659 (5.9) |
|  |  |  |  |
| Space rental and facility fee | N/A | $13,493,886 (5.7) | $13,493,886 (5.7) |
|  |  |  |  |
| Direct payments | $1,467,242 (0.6) | $11,575,332 (4.9) | $13,042,574 (5.5) |
| Gift | $44,354 (0.0) | $7,920,033 (3.4) | $7,964,387 (3.4) |
| Charitable contribution | $500.00 (0.0) | $3,419,000 (1.4) | $3,419,500 (1.4) |
| Consulting fee | $808,437 (0.3) | $174,086 (0.1) | $982,523 (0.4) |
| Royalty or license | $613,951 (0.3) | $62,214 (0.0) | $676,165 (0.3) |
|  |  |  |  |
| Research | N/A | $3,296,916 (1.4) | $3,296,916 (1.4) |
|  |  |  |  |
